# Factors associated with mental distress among undergraduate students in northern Tanzania

**DOI:** 10.1186/s12888-020-2448-1

**Published:** 2020-01-29

**Authors:** Innocent B. Mboya, Beatrice John, Eneck S. Kibopile, Lisbeth Mhando, Johnston George, James S. Ngocho

**Affiliations:** 1 Institute of Public Health, Community Health Department, Kilimanjaro Christian Medical University College (KCMUCo), P. O. Box 2240, Moshi, Tanzania; 2 Institute of Public Health, Department of Epidemiology and Biostatistics, Kilimanjaro Christian Medical University College (KCMUCo), P. O. Box 2240, Moshi, Tanzania; 3 Institute of Public Health, Department of Behavioral and Social Sciences, Kilimanjaro Christian Medical University College (KCMUCo), P. O. Box 2240, Moshi, Tanzania; 4 School of Mathematics, Statistics & Computer Science, University of KwaZulu-Natal, Pietermaritzburg, Private Bag X01, Scottsville, 3209 South Africa

**Keywords:** Mental distress, Mental health, Mental illness, Undergraduate students, Tanzania

## Abstract

**Background:**

Mental distress is a major public health problem which includes anxiety, depression and somatic symptoms such as sleeping problems, fatigue and headache. University students are consistently reported to have higher levels of mental distress compared to the general population. Although university students with mental distress have significantly impaired cognitive functioning, learning disabilities and poor academic performance, the burden of this problem in Tanzania is unknown. This study aimed to determine prevalence and factors associated with mental distress among undergraduate students in northern Tanzania.

**Methods:**

A cross-sectional study was conducted among undergraduate students at Kilimanjaro Christian Medical University College from April–July 2018. Simple random sampling technique using probability proportional to size was used to sample students from their respective classes. Mental distress was screened using the self-reporting questionnaire (SRQ-20). Data was analyzed using Stata version 15.1. Frequencies and percentages were used to summarize categorical variables while mean and standard deviation for numeric variables. Multivariable logistic regression was used to determine factors associated with mental distress adjusted for potential confounders.

**Results:**

A total of 402 undergraduate students participated in this study, 14% screened positive for mental distress. Residing off-campus (OR = 0.44, 95%CI 0.20–0.96) and perceived availability of social support (OR = 0.22, 95%CI 0.11–0.45) reduced the odds of mental distress while students with family history of mental distress (OR = 2.60, 95%CI 1.04–6.57) and those with decreased grades than anticipated (OR = 3.61, 95%CI 1.91–6.83) had higher likelihood of mental distress.

**Conclusion:**

One in every ten students screened was positive for mental distress. Those who reported a family history of mental illness and lower grades than anticipated had higher response of mental distress. To relieve students from stress and frustrations related to studies and their lives in general, this study recommends awareness creation, counselling to help those with mental health issues, establishment of student drop-in centers for such services and promotion of social and recreational activities at the college.

## Background

Mental distress is a major public health problem that affect society as a whole and it includes mental problems such as anxiety, depression and somatic symptoms for example sleeping problems, fatigue, headache and back ache [1, 2]. University students are consistently reported to have higher levels of mental distress compared to the general population [1–4]. For instance, significantly higher levels of mental distress were reported among Australian medical students as compared to the general population [4, 5].

Higher prevalence of mental distress among university students have also been reported in Asian and sub-Saharan Africa countries [3, 6–10]. The highest observed proportion was 71.9% among medical students in Jizan University, Kingdom of Saudi Arabia [11] which is almost similar to that reported in Tanzania (70%) among non-medical students in Dar Es Salaam [12]. Mental health problems such as mental distress are not well documented in Tanzania. The proportions of mental distress among undergraduate students in medical institutions in other studies are high, which may also be true among students in Tanzania. Data from this population is crucial in promoting health and well-being in the student population [13].

University students with higher proportion of mental distress are likely to experience negative consequences such as significant impaired cognitive functioning, learning disabilities, poor academic performance, substance abuse (e.g. cigarette/tobacco smoking, alcohol use and khat chewing) which are associated with risk behaviors [8, 14], higher risk of depression as well as anxiety disorders [8]. This indicates that, mental distress increases the risk of other mental health problems.

Mental distress among university students has been associated with several factors such as sex (i.e. female students reporting higher levels compared to males), lack of interest towards the field of study, not having close friends, never attending religious programs, conflict with friends, financial problems, family history of mental illness, use of drugs such as khat, lack of break or vacation, limited social support, examinations, very tight schedules and lack of extracurricular activities around the campus [1, 5, 10, 11].

Tanzania, as other low- and middle-income countries faces many challenges in meeting mental health needs, being among one of the least prioritized areas. These include inadequate number of trained personnel in mental health, misplacement of human resource for mental health, lack of specific treatment, problematic insurance coverage for mental disorders, and stigma attached to mental health problems [15]. Due to these challenges, mental health problems may be intensified especially in key populations such as university students. This study aimed at determining prevalence and factors associated with mental distress among undergraduate students in northern Tanzania.

## Methods

### Study design and setting

This cross-sectional study was conducted from April–July 2018 at Kilimanjaro Christian Medical University College (KCMUCo). KCMUCo is among the four universities in Moshi Municipality, northern Tanzania and is the only university in the region offering medical education and other allied health programs. Programs offered in this higher learning institution includes Diploma and Bachelor’s in health laboratories, Bachelor in Nursing, Diploma in Occupation Therapy, Diploma and Bachelor in Physiotherapy, Diploma and Bachelor in Prosthetics and Orthotics, Doctor of Medicine, Bachelor in Optometry and Diploma in HIV and AIDS care. The college had a total of 1100 undergraduate students in the year 2018.

### Study population and sample size

The study population was undergraduate students at KCMUCo who were in class during the data collection period and provided their informed consent. A single proportion formula was used for sample size calculation using a standard normal value of 1.96 at 95% confidence interval, 5% margin of error and an estimated prevalence of mental distress among undergraduate students of the University of Gondar University in Ethiopia (40.9%) [1]. Adding 10% of non-response, the minimum required sample size was estimated to be 410 participants. Simple random sampling technique using probability proportional to size was used to sample students from their respective classes.

### Study variables

The dependent variable was mental distress which was measured using a self-reporting questionnaire (SRQ-20). The SRQ-20 is a standardized questionnaire having 20 item questions, originally developed by World Health Organization (WHO) to indicate mental distress [1, 16]. The SRQ-20 has been validated in low- and middle-income countries and found to be highly sensitive and specific [16–21]. The tool has also been used among university students in several studies in African settings [1, 8, 9]. Students in this study were asked if they experienced the SRQ-20 symptoms within 1 month preceding the survey. Those with a score of 8 or more were considered having mental distress [1, 9]. Independent variables included; background characteristics such as age in years, sex, year of study, area of residence (on-campus and off-campus) and smoking status (ever smoking tobacco, marijuana or both). Family history of mental illness, history of alcohol use, lack of break or vacation, increased class work load, decreased grades than anticipated and missing too many classes were measured as binary (yes and no). Perceived social support was measured using a 12 items social support questionnaire (SSQ) with responses ranging from strongly agree-1 to strongly disagree-5 [22]. After performing principal component analysis (PCA), 11 out of 12 statements predicted the first component. The remaining statements were added and divided by 11 to compute social support variable. The values obtained ranged from 1 to 4.9. Participants with 1 to 2 values were categorized as having social support. Statements and results used to generate social support variable are provided in the Additional file 1.

### Data collection

Students were given a questionnaire to fill-out (see Additional file 2). The questionnaire was in English, an official language at higher learning institutions in Tanzania. Data collection was performed by three trained medicine students at KCMUCo. Data collectors visited undergraduate students in their respective classes and clearly explained the purpose of the study. Once all questions or concerns related to the study were addressed, students were sampled proportional to the size of each class and were administered informed consent. Following a written consent, students were given a questionnaire to complete which took a maximum of 25 min. The trained student data-collectors did not also complete a survey. Finally, all the completed questionnaires were collected and reviewed for completeness.

### Statistical analysis

Data was entered and cleaned using Statistical Package for Social Science (SPSS) (SPSS Inc., Chicago, IL) version 21 and analyzed using STATA® software (Stata Corp LP) version 15.1. Frequency and percentages were used to describe prevalence of mental distress. Means and standard deviations were used to summarize numeric variables. Cronbach’s alpha was used to assess reliability of the 12 statements used to measure availability of social support that provided a scale of 88.6%. Principal component analysis was used to extract a total of 11 out of 12 statements that measured availability of social support in this study. From these, social support was computed as a binary variable.

Odds ratios (ORs) with 95% confidence intervals (CIs) for factors associated with mental distress were estimated using a multivariable logistic regression model. Stepwise logistic regression was used to select variables to be included in the multivariable model at 10% significance level. Likelihood ratio test was used to compare a model with and without interaction providing evidence for a later model. A *p*-value of less than 5% was considered statistically significant.

## Results

### Background characteristics of study participants

The study enrolled a total of 402 undergraduate students at KCMUCo. The mean age of study participants was 24 years and standard deviation of 2.4 years. Half of participants (50.5%) were pursuing medicine program while nursing students accounted for 22% of the sample. Of all respondents, 92.8% received government funding through higher education student loans board scheme. The proportion of students who reported family history of mental illness was 7.5% while 53.2% reported availability of social support system when needed (Table 1).

**Table 1:** Background characteristics of study participants (*N* = 402)

Variable	n	%
Age (years)		
Mean (SD)	24 (2.4)	
20–24	281	69.9
25+	121	30.1
Sex		
Male	243	60.4
Female	159	39.6
Place of residence		
On-campus	60	14.9
Off-campus	342	85.1
Year of study		
Second year	127	31.6
Third year	175	43.5
Fourth and fifth years	100	24.9
Program of study		
Doctor of medicine	203	50.5
Nursing	88	21.9
Others^a^	111	27.6
Source of funding		
Family/personal	21	5.2
HESLB	373	92.8
Sponsor	8	2
Marital status		
Single	358	89.0
Married/ Cohabiting	44	11.0
Have pocket money		
No	115	28.6
Yes	287	71.4
Reported family history of mental illness		
No	372	92.5
Yes	30	7.5
Ever drank alcohol		
No	290	72.1
Yes	112	27.9
Ever smoked tobacco or marijuana		
No	359	89.3
Yes	43	10.7
Social support available		
No	188	46.8
Yes	214	53.2

^a^Other programs of study included; laboratory sciences (14.2%), physiotherapy (7.0%), prosthetics and orthotics (3.7%) and optometry (2.7%). HESLB, Higher Education Students’ Loans Board

### Reported academic challenges

The most common academic challenges reported among study participants were: increased class workload (33.1%), lack of vacation or break (37.3%) and decreased grade than anticipated (21.1%) (Table 2).

**Table 2:** Reported academic challenges

Variable	Frequency	Percentage
Increase class work load		
No	269	66.9
Yes	133	33.1
Decreased grade than anticipated		
No	317	78.9
Yes	85	21.1
Missed too many classes		
No	362	90.0
Yes	40	10.0
Serious argument with instructors		
No	370	92.0
Yes	32	8.0
Lack of vacation/ break		
No	252	62.7
Yes	150	37.3

### Proportion of students screened positive for mental distress

The distribution of questions used to measure mental distress are shown in Table 3. Among 402 students who participated in this study, 56 (13.9%) screened positive for mental distress.

**Table 3:** Symptoms of mental distress (*N* = 402)

Symptoms of mental distress^a^	Frequency^b^	Percentage^b^
Often have headache	106	26.4
Poor appetite	61	15.2
Lack of sleep	64	15.9
Easily frightened	61	15.2
Uncontrollable hand shaking	37	9.2
Feeling nervous	67	16.7
Poor digestion	49	12.2
Trouble on thinking	40	10
Feeling unhappy	69	17.2
Crying more than usual	23	5.7
Difficult to enjoy daily activities	57	14.2
Daily work suffering	41	10.2
Unable to play a useful part in life	43	10.7
Lost interest in things	92	22.9
Feeling of worthlessness	38	9.5
Thought of ending life	43	10.7
Feeling tired all the time	50	12.4
Uncomfortable feeling in the stomach	71	17.7
Difficult to make decisions	59	14.7
Easily tired	66	16.4
Mental distress		
Yes	56	13.9
No	346	86.1

^a^Symptoms based on self-reporting questionnaire (SRQ-20) originally developed by WHO. ^b^Frequency and percentage among those who answered “Yes”

### Factors associated with mental distress

Factors found to be significantly associated with mental distress in the crude analysis were year of study, family history of mental illness, availability of social support, increased class workload, deceased grade than anticipated, missing too many classes and lack of vacation (Table 4).

**Table 4:** Crude odds ratio for factors associated with mental distress

Characteristics	n	Have mental distress (%)	COR^a^ (95%CI)	*p*-value	AOR^b^ (95%CI)	*p*-value
Age (years)						
20–24	281	40 (14.2)	1			
25+	121	16 (13.2)	0.92 (0.49–1.71)	0.79	–	
Sex						
Male	243	32 (13.2)	1			
Female	159	24 (15.1)	1.17 (0.66–2.08)	0.59	–	
Residence						
On-campus	60	13 (21.7)	1		1	
Off-campus	342	43 (12.6)	0.52 (0.26–1.04)	0.06	0.44 (0.20–0.96)	0.04
Year of study						
Second year	127	24 (18.9)	1			
Third year	175	16 (9.1)	0.43 (0.22–0.85)	0.02	**–**	
Fourth and fifth years	100	16 (16.0)	0.82 (0.41–1.64)	0.57	–	
Reported family history of mental illness						
No	372	46 (12.4)	1		1	
Yes	30	10 (33.3)	3.54 (1.56–8.04)	0.002	2.60 (1.04–6.57)	0.04
Ever drank alcohol						
No	290	40 (13.8)	1			
Yes	112	16 (14.3)	1.04 (0.56–1.95)	0.90	–	
Ever smoked tobacco or marijuana						
No	358	46 (12.8)	1			
Yes	43	10 (23.3)	2.06 (0.95–4.46)	0.07	2.37 (1.00–5.59)	0.05
Social support available						
No	188	43 (22.9)	1		1	
Yes	214	13 (6.1)	0.22 (0.11–0.42)	< 0.001	0.22 (0.11–0.45)	< 0.001
Increase class work load						
No	269	28 (10.4)	1			
Yes	133	28 (21.1)	2.30 (1.29–4.07)	0.004	**–**	
Decreased grade than anticipated						
No	317	30 (9.5)	1		1	
Yes	85	26 (30.6)	4.22 (2.32–7.65)	< 0.001	3.61 (1.91–6.83)	< 0.001
Missed too many classes						
No	362	45 (12.4)	1			
Yes	40	11 (27.5)	2.67 (1.25–5.72)	0.01	**–**	
Serious argument with instructors						
No	370	48 (13.0)	1			
Yes	32	8 (25.0)	2.24 (0.95–5.26)	0.07	–	
Lack of vacation/ break						
No	252	26 (10.3)	1			
Yes	150	30 (20.0)	2.17 (1.23–3.84)	0.008	**–**	

^a^COR – Crude odds ratio. ^**b**^*AOR* Adjusted odds ratio – adjusted for area of residence, family history of mental illness, availability of social support, decreased grade than anticipated, alcohol use and smoking status

In the multivariable logistic regression analysis, stepwise regression was fit including variables that were statistically significant in the crude analysis. Age and sex were considered as potential confounders though results from the crude analysis did not show a significant association with mental distress. Likelihood ratio test was used to assess the effect of including or excluding age and sex in the final model which showed no statistically significant effect (*p* > 0.05) of including them in the final model. Therefore, the final multivariable model indicated that, student’s area of residence, family history of mental illness, perceived availability of social support and decreased grade than anticipated were the factors significantly (*p* < 0.05) associated with mental distress in this population (Table 4). Residing off-campus (OR = 0.44, 95%CI 0.20–0.96) and perceived availability of social support (OR = 0.22, 95%CI 0.11–0.45) reduced the odds of mental distress while higher odds of mental distress were found among students with family history of mental distress (OR = 2.60, 95%CI 1.04–6.57) and those with decreased grades than anticipated (OR = 3.61, 95%CI 1.91–6.83).

## Discussion

This study aimed to determine prevalence and factors associated with mental distress among undergraduate students in northern Tanzania. About 14% of undergraduate students in this population screened positive for mental distress. Area of residence, family history of mental illness, availability of social support and decreased grade than anticipated were the factors found to be significantly associated with mental distress.

Prevalence of mental distress among undergraduate students in this study is similar to that reported in France (12.6%) [23]. However, our estimate is lower than those reported in Ethiopia (29.2–41%) [1, 2, 9] and Somalia (19.8%) [8]. Significant differences observed in Ethiopia can be explained by the scoring of the items where in this study the scores ranged from 1-strongly agree to 5-strongly disagree as compared to [1] whose scores ranged from 1-very strongly disagree to 7-very strongly agree. The study also had a larger sample size compared to the current study. Moreover, other studies included first year students who are likely to face difficulties in adapting to the university environment but were not available to participate in this study. James et al., [3] assessed perceived medical students stress but used scores instead of a binary categorical variable that estimates prevalence/proportion as done in this study. A qualitative study in UK also reported that many of the students were suffering from mental distress whereby stigma attached to this issue intensifies the problem [13].

Lower odds of mental distress were observed among students who were residing off-campus. It is possible that, those who were residing on-campus were probably spending more time studying compared to those staying off-campus. While the later could also be using college premises to study, they are forced to leave earlier due to safety issues [9]. In Australia, accommodation circumstances (such as living in rental houses as opposed to family or own homes) and longer travel times increased the likelihood of having mental distress [5]. Only 15 % of students in this study were residing on-campus. College accommodation should therefore be promoted as one of the potential strategies to support students manage stress.

Availability of social support among undergraduate students in this study reduced the odds of mental distress as also reported in Ethiopia [1]. This implies that, having available support from both friends and family members reduces stress associated with studies and adjusting to the college environment. Not having a satisfying relationship with the family increased the risk of mental distress in Somalia and Ethiopia [2, 8]. There is a need to encourage university students to establish healthy relationships with both their families, friends and fellow students in order to reduce the burden of this problem. Student support services at the college should also be strengthened such as using peer counselors who will play role in addressing and eventually reducing mental health issues among undergraduate students.

Furthermore, similar to a study in Ethiopia [1], family history of mental illness significantly increased the odds of mental distress in this study. This may be explained by genetic predisposition in addition to challenges associated with caring for a mentally ill family member [1, 24]. Students with family history of mental illness may benefit from counselling services if they are made available both at the college and the surrounding community environment.

The amount of stress is likely to increase among university students because of a high parental expectation, very tight academic program and during examinations period which also increases after receiving unexpectedly low scores [3, 10, 11]. In this study, students experiencing decreased grades than anticipated had higher odds of mental distress. This could imply that, university students are likely to experience depression, anxiety, frustration and troubles sleeping among other mental health issues due to stress associated with their academic lives [14, 23, 25].

The current study is the first to determine the burden of mental distress among undergraduate university students in Tanzania. This study may have been prone to information bias whereby students might have misreported information related to different symptoms of mental distress as well as engagement in risk behaviors such as alcohol and drug use.

## Conclusions

Prevalence of mental distress was found to be 14%. Factors associated with mental distress among undergraduate students in this study are area of residence, availability of social support, family history of mental illness and decreased grades than anticipated. This study recommends awareness creation as well as counselling to help students with mental health issues such as mental distress. Universities and colleges should consider establishing student drop-in centers that will provide counselling services to students experiencing different mental health issues. Social and recreational activities at the college can play a significant role in relieving students from stress and frustrations related to studies and their lives in general.

## Supplementary information

**Additional file 1:** **Table S1.** Perceived availability of social support.

**Additional file 2.** Questionnaire used to assess prevalence and factors associated with mental distress among undergraduate students at KCMUCo.

## Data Availability

The datasets used and/or analyzed during the current study are available from the corresponding author on reasonable request.

## Abbreviations

KCMUCo: Kilimanjaro Christian Medical University College
OR: Odds Ratio
SRQ-20: Self-Reporting Questionnaire-20
