# Supplementary material for: Factors associated with mental distress among undergraduate students in northern Tanzania
Source: BMC Psychiatry. 2020 Jan 29;20:28. doi: 10.1186/s12888-020-2448-1 (PMC6988278; doi:10.1186/s12888-020-2448-1)
Supplement: Supplementary file 1 — Additional file 1: Table S1. Perceived availability of social support. [file 12888_2020_2448_MOESM1_ESM.docx]

## Table S1: Perceived availability of social support

| **Statements** | **Strongly agree**  **n (%)** | **Agree**  **n (%)** | **Neutral**  **n (%)** | **Disagree**  **n (%)** | **Strongly disagree**  **n (%)** |
| --- | --- | --- | --- | --- | --- |
| There is a special person who is around when I am in need | 179 (44.5) | 116 (28.9) | 71 (17.7) | 15 (3.7) | 21 (5.2) |
| There is a special person with whom I can share my joys and sorrows | 165 (41.0) | 134 (33.3) | 69 (17.2) | 19 (4.7) | 15 (3.7) |
| There is a special person in my life who cares about my feelings | 152 (37.8) | 118 (29.4) | 89 (22.1) | 28 (7.0) | 16 (3.7) |
| I have a special person who is a real source of comfort to me | 151 (37.6) | 126 (31.3) | 81 (20.2) | 21 (5.2) | 23 (5.7) |
| I have friends with whom I can share my joys and sorrows | 134 (33.3) | 148 (36.8) | 79 (19.7) | 23 (5.7) | 18 (4.5) |
| I get emotional help and support I need from my family | 205 (51.0) | 104 (25.9) | 55 (13.7) | 23 (5.7) | 15 (3.7) |
| My friends really try to help me | 120 (29.9) | 155 (38.6) | 90 (22.4) | 22 (5.5) | 15 (3.7) |
| My family really tries to help me | 239 (59.5) | 110 (27.4) | 27 (6.7) | 15 (3.7) | 11 (2.7) |
| I can count on my friends when things go wrong | 106 (26.4) | 151 (37.6) | 91 (22.6) | 35 (8.7) | 19 (4.7) |
| I can talk about my problems with my family | 155 (38.6) | 127 (31.6) | 80 (19.9) | 29 (7.2) | 11 (2.7) |
| I can talk about my problems with my friends | 105 (26.1) | 157 (39.1) | 92 (22.9) | 29 (7.2) | 19 (4.7) |
| My family is willing to help me make decisions | 159 (39.6) | 142 (35.3) | 60 (14.9) | 25 (6.2) | 16 (4.0) |

Note: 1=Strongly agree, 2=Agree, 3=Neutral, 4=Disagree and 5=Strongly disagree
