# Supplementary material for: Factors associated with mental distress among undergraduate students in northern Tanzania
Source: BMC Psychiatry. 2020 Jan 29;20:28. doi: 10.1186/s12888-020-2448-1 (PMC6988278; doi:10.1186/s12888-020-2448-1)
Supplement: Supplementary file 2 — Additional file 2. Questionnaire used to assess prevalence and factors associated with mental distress among undergraduate students at KCMUCo. [file 12888_2020_2448_MOESM2_ESM.pdf]

Kilimanjaro Christian Medical University College

Department of Community Health, Institute of Public Health

P.O.Box 2240, Moshi – Tanzania. Website; http://kcmuco.ac.tz/. Email: info@kcmuco.ac.tz

Study Title: Prevalence and factors associated with mental distress among undergraduate students in northern Tanzania

Questionnaire

Part I. Socio-economic and demographic characteristics of the respondents

This part of the questionnaire assesses about your socio economic and demographic information's, after reading the following the questions please give appropriate answer concerning your socio-economic and demographic information.

No	Questions	Response/Options	Remarks
1.	Age in years	______________	
2.	Sex	1. Male<br>2. Female	
3.	Residence	1. Urban<br>2. Rural	
4.	Faculty choice	1. Preferred<br>2. Not preferred	
5.	Year of study	1. 1st year<br>2. 2nd year<br>3. 3rd year<br>4. 4th year<br>5. 5th year	
6.	Females: Do you have boyfriend	1. Yes<br>2. No	
7.	Males: Do you have girlfriend	1. Yes<br>2. No	
8.	Marital Status	1. Not married<br>2. Married<br>3. Cohabiting<br>4. Divorced	
9.	How often do you take part in religious practices	1. Always<br>2. Often<br>3. Some times<br>4. Never	
10.	How often do you face conflict with your friends in the dormitories/class rooms	1. Always<br>2. Often<br>3. Some times<br>4. Never	

11.	Do you have pocket money?	1. Yes<br>2. No	If No, go to Q13
12.	If yes for question no 11, what is your monthly amount of pocket money in Tanzanian shillings?	___________	
13.	In your family, is there a history of mental illness?	1. Yes<br>2. No	

Part II. Mental distress Self-Reporting Questionnaire

The following questions are related to certain pains and problems that may have bothered you in the last **30 days**. Please tick the **'Yes'** box if you have had this symptom in the last 30 days. On the other hand, if you have not experienced this symptom in the last 30 days please tick the **'No'** box. 0= No, 1= Yes.

Please tick the **'yes** box if you have had experienced these symptoms in the **last 30 days** and tick the **'no** box if you had not**.**			
1.	Do you often have headaches?	☐No	☐ Yes
2.	Is your appetite poor?	☐ No	☐ Yes
3.	Do you sleep badly?	☐ No	☐ Yes
4.	Are you easily frightened?	☐ No	☐ Yes
5.	Do your hands shake?	☐ No	☐ Yes
6.	Do you feel nervous?	☐ No	☐ Yes
7.	Is your digestion poor?	☐ No	☐ Yes
8.	Do you have trouble thinking clearly?	☐ No	☐ Yes
9.	Do you feel unhappy?	☐ No	☐ Yes
10.	Do you cry more than usual?	☐ No	☐ Yes
11.	Do you find it difficult to enjoy your daily activities?	☐ No	☐ Yes
12.	Do you find it difficult to make decisions?	☐ No	☐ Yes
13.	Is your daily work suffering?	☐ No	☐ Yes
14.	Are you unable to play a useful part in life?	☐ No	☐ Yes
15.	Have you lost interest in things?	☐ No	☐ Yes
16.	Do you feel that you are a worthless person?	☐ No	☐ Yes
17.	Has the thought of ending your life been on your mind?	☐ No	☐ Yes
18.	Do you feel tired all the time?	☐ No	☐ Yes
19.	Do you have uncomfortable feelings in the stomach?	☐ No	☐ Yes
20.	Are you easily tired?	☐ No	☐ Yes

PART III. Substance use

The following questions focuses on marijuana Khat chewing practices, Alcohol drinking, Cigarette smoking other substances like Heroin use and sedatives use. So, you are kindly requested to give a genuine answer about your personal behaviour on the use of these substances.

Please tick the **‘yes** box if you have had use and **‘no** box if you don’t use the mentioned substance in your life time or in the last 1 month as directed. 0= No, 1= Yes			
1.	Have you ever used marijuana in your life?	☐ No	☐ Yes
2.	Have you used marijuana in the last 1 month?	☐ No	☐ Yes
3.	Have you ever used alcohol drinks (like beer or any other alcohol drinks) in your life time	☐ No	☐ Yes
4.	Have you used any kind of alcohol drinks in the last one month?	☐ No	☐ Yes
5.	Have you ever used Tobacco products such as cigarette smoking, shisha any other tobacco products in your life time?	☐ No	☐ Yes
6.	Have you used any kind of tobacco product in the last one month?	☐ No	☐ Yes
7.	Have you ever used other substances such as khat, heroin, cocaine, cannabis or others?	☐ No	☐ Yes
8.	Have you used any other substances such as khat, heroin, cocaine, cannabis or others in the last one month?	☐ No	☐ Yes
9.	Have you ever injected drugs?	☐ No	☐ Yes
10.	If Yes in Q9 above, In the past one month, have you injected drugs	☐ No	☐ Yes

Part IV. Academic problems

This part assesses academic problems that students face/experience in the course of their studies. The answer is provided by **“Yes”** or **“No”.**

Please tick the **‘lease** box if you have experienced and **‘ox i**nbox if you don’t in the last academic year.			
1.	Increase class work load	☐ No	☐ Yes
2.	Decrease grade than anticipated	☐ No	☐ Yes
3.	Missed too many class	☐ No	☐ Yes
4.	Serious arguments with instructors	☐ No	☐ Yes
5.	Lack of vacations /break	☐ No	☐ Yes

Part V: Social Support

This part assesses about social support that you have got from your family, friends and significant others. Each item is scored 1= strongly agree, 2= Agree, 3=Neutral, 4= Disagree, 5= Strongly disagree. Circle the appropriate response.

		Strongly agree	agree	neutral	disagree	Strongly disagree
1.	There is a special person who is around when I am in need	1	2	3	4	5
2.	There is a special person with whom I can share my joys and sorrows	1	2	3	4	5
3.	My family really tries to help me	1	2	3	4	5
4.	I get emotional help and support I need from my family	1	2	3	4	5
5.	I have a special person who is a real source of comfort to me	1	2	3	4	5
6.	My friends really try to help me	1	2	3	4	5
7.	I can count on my friends when things go wrong	1	2	3	4	5
8.	I can talk about my problems with my family	1	2	3	4	5
9.	I have friends with whom I can share my joys and sorrows	1	2	3	4	5
10.	There is a special person in my life who cares about my feelings	1	2	3	4	5
11.	My family is willing to help me make decisions	1	2	3	4	5
12.	I can talk about my problems with my friends	1	2	3	4	5

Thank you very much for your participation!
